# Concept Development for Bearing Fault Detection on Water-Cooled Electric Machines Using Infrared

**DOI:** 10.3390/s25072170

**Published:** 2025-03-29

**Authors:** Stephanie Schamberger, Lukas Brandl, Hans-Christian Reuss, Alfons Wagner

**Affiliations:** 1 Research Institute for Automotive Engineering and Powertrain Systems Stuttgart (FKFS), 70569 Stuttgart, Germany; stephanie.schamberger@fkfs.de (S.S.); alfons.wagner@fkfs.de (A.W.); 2 Department of Automotive Mechatronics, Institute of Automotive Engineering (IFS), Faculty 7: Engineering Design, Production Engineering and Automotive Engineering (F07), University of Stuttgart, 70569 Stuttgart, Germany

**Keywords:** infrared technology, thermography, water-cooled EM

## Abstract

Electric machines (EMs) of electrified vehicle drivetrains can be tested on drivetrain test benches at an early stage of development. In order to protect the EMs from premature damage or failure during testing, monitoring their thermal condition is important. Due to the package requirements of compact and powerful EMs with high-speed requirements and high-power densities, the heat build-up inside the motor during operation is particularly high. For this reason, fluid cooling with heat exchangers is increasingly being used in EMs. The EMs analysed in this work are water-cooled by a cooling jacket. This influences the heat flow inside the machine through heat transfer mechanisms, making it difficult to detect damage to the EMs. This paper presents a novel method for non-destructive and non-contact thermal condition monitoring of water-cooled EMs on drivetrain test benches using thermography. In an experimental setup, infrared images of an intact water-cooled EM are taken. A bearing of the EM’s rotor is then damaged synthetically, and the experiment is repeated. The infrared images are then processed and analysed using appropriate software. The analysis of the infrared images shows that the heat propagation of the motor with bearing damage differs significantly from the heat propagation of the motor without bearing damage. This means that thermography opens up another method of condition monitoring for water-cooled EMs. The results of the investigation serve as a basis for future condition monitoring of water-cooled EMs on powertrain test benches using artificial intelligence (AI).

## 1. Introduction

Development time plays a decisive role in today’s vehicle development. In order to reduce costs, the development time for vehicles is becoming ever shorter. As a result, the individual components are tested as prototypes before the entire vehicle is tested. In vehicle technology, the individual components are tested on hardware-in-the-loop (HiL) test benches. The hardware to be tested includes EMs, gearboxes and engines. The rest of the vehicle is simulated in the real environment. The thermal, mechanical and hydrodynamic properties of the components are analysed on HiL test benches at a very early stage of development. Both initial commissioning and endurance testing of the prototypes can be carried out on these test benches. The knowledge gained from testing is used to identify deficiencies in the prototypes and thus serve the further development of the prototypes. Testing on HiL test benches and vehicle tests with prototypes can reduce development time and costs and minimise development risks [1]. Accordingly, the testing of components on test benches is an indispensable part of vehicle development. This work includes the testing of EMs on test benches. Modular and highly specialised HiL test benches are required to carry out such testing on EMs. The EMs to be tested are valuable as they are prototypes, and only very small quantities are available. As a result, these EMs are expensive and their condition monitoring during testing is of particular interest in order to avoid mechanical and thermal damage. A wide range of measurement technology is used to monitor the EMs in order to check them and prevent failures. Among other things, measurement technology is used to monitor vibration velocity, speed and torque. By monitoring the actual state of the EMs, the smallest deviations from the desired standard state of the EMs are registered. This enables damage to be recognised at an early stage. In addition to monitoring the vibration velocities, temperature monitoring plays a particularly important role. An unexpected change in temperature can indicate bearing damage or a winding fault at an early stage. As the EMs in the field of vehicle technology have a water- or oil-cooled cooling jacket, thermal monitoring is significantly more difficult compared to air-cooled EMs. The water-cooled cooling jacket conceals the spread of heat inside the EM. The heat generated is dissipated directly through the coolant. The EM also has an additional jacket. As a result, it is not possible to determine the temperature inside the motor from the outside in a non-destructive manner. The temperatures are usually monitored by PT 100 temperature sensors, for which the EMs have to be machined. The surface temperature is usually measured using type K thermocouples, which are glued to the surface of the EMs, but this requires access to the machine. Infrared thermography, on the other hand, is suitable for holistic temperature measurement. Infrared measuring devices are used for this purpose, which record the infrared radiation emitted by an object and convert it into an electronic signal [2]. For temperature monitoring on rotating machines, thermography offers several advantages over the previously mentioned methods. Among other things, thermography enables non-invasive, high-precision measurement and visualisation of the surface temperature of the EM [3]. While the measuring range of thermocouples is limited to a few physical measuring spots, the measuring range can be significantly increased by using thermography. Each pixel represents a temperature measuring point, which corresponds to 307,200 measuring points for the thermal imaging camera used in this work, for example. This means that the surface of the EMs can be monitored for temperature changes. Based on these advantages of thermography for determining the temperature of EMs, several scientists have already dealt with this topic. Accordingly, Alvarado-Hernandez et al. [4] utilised thermography not only for EMs but even for multiple fault diagnosis on an entire kinematic chain. Trejo-Chavez et al. [5] used thermography for the Convolutional Neural Network (CNN)-based identification of mechanical faults, such as bearing damage and broken rotor bars in induction motors. How the detection of hotspots, the subsequent calculation of the efficiency and the resulting fault detection of induction motors is carried out is presented in the work of Badoni and Jarial [6]. The work by Munoz-Ornelas et al. [7] refers to the effect of the camera location for thermography on induction motors. The fact that thermography is also of interest for fault detection based on machine learning is explained in the work of Atif et al. [8]. Accordingly, there is still a great need for scientific investigations of induction motors using thermography. The purpose of this article is to present an innovative approach for the application of thermography to water-cooled EMs for bearing damage detection. This new approach utilises the proven measurement technique of thermography, which is now applied to EMs with a water-cooled jacket. The challenge is that the heat flow inside the machine is difficult to determine due to the cooling jacket. The surface temperature on the motor housing surface does not correspond to the temperature inside the motor due to the additional jacket and the cooling water between the motor and the housing surface. The heat generated inside the motor is dissipated through the cooling jacket so that the heat is not visible on the surface of the housing [9]. This means that a hotspot inside the EM cannot be detected. Unnoticed hotspots, e.g., in bearings, can lead to bearing damage, which can cause the EM to fail. This paper presents a new method that can be used to detect hotspots on the EM despite the cooling jacket. The following section begins with an introduction to the thermal processes of water-cooled EMs and the basics of thermography. This is followed by a brief overview of previously published work on the condition monitoring of air-cooled EMs using thermography. This is followed by a presentation of the method. The method includes a test setup with the resulting measurement data. After the presentation of the method, the results of the measurements are discussed.

## 2. Theoretical Background

### 2.1. Thermal Processes in Electric Machines

Thermal monitoring plays a major role in the testing of EMs on test benches. In order to recognise damage in good time and avoid EM failures, thermal monitoring of the EMs is absolutely essential. In order to gain a better understanding of the thermal processes in EMs, this is discussed below. The operation of EMs generates energy losses that heat the windings, the magnetic circuits and the magnetically inactive parts of the machine [10]. The lossy components in EMs are the stator laminations, the windings, the rotor laminations, the bearings of the rotor shaft and the permanent magnets in the rotor. Air friction also occurs inside the machine. The total energy loss is caused by the following loss mechanisms: copper losses, iron losses, permanent magnet losses, bearing friction losses and air friction losses. The torque in the base speed range is approximately proportional to the amount of current, to which the copper losses increase quadratically. Friction and internal losses, on the other hand, are disproportionately dependent on the frequency. This means that copper losses occur almost exclusively in low-speed ranges. In total, significantly higher losses occur in the stator than in the rotor [9]. Due to the temperature resistance of the winding insulation, the temperature requirements in EMs are limited. The prescribed maximum temperature of the electric insulation must not be exceeded during testing. Exceeding the maximum temperature can lead to rapid ageing or overall failure, which can result in a short circuit and unbalanced stator windings, for example, the protective varnish on the windings melts. The maximum temperatures are between 120 and 160 °C. Motor performance is also affected by a high operating temperature, as the electric resistance in the copper windings increases and the magnetic properties of the permanent magnets change. These can demagnetise at high temperatures [11].

Cooling is necessary to prevent damage to the components that are heated by energy loss. The EMs can be cooled externally via the motor housing using jacket cooling. However, the effectiveness of the cooling is reduced as the active components are only cooled indirectly. Liquid media, such as glycol–water mixtures, or gases are used for jacket cooling [11]. The coolant is channelled through the circumferential cooling channels between the stator and motor housing. The heated liquid is fed into the cooling circuit via a water nozzle at the end of the cooling jacket [12].

The basic heat transfer mechanisms involved inside EMs are thermal conduction, convection and thermal radiation. Thermal conduction is the diffused transport of energy between neighbouring molecules in fluids or solids due to a temperature gradient. Heat transfer in a flowing fluid is referred to as convection. The calculation of one-dimensional, convective heat transfer $\dot{Q}$ is performed as in Equation (1). The thermal resistance is described by Equation (2) [9]:

(1) $$\dot{Q}=\alpha A_{\mathrm{ben}}(\vartheta_{\mathrm{Fl}}-\vartheta_{\mathrm{Wa}})$$

(2) $$R_{\mathrm{th},\mathrm{conv}}=\frac{1}{\alpha A_{\mathrm{ben}}}$$

αHeat transfer coefficientW/(m^2^K)$A_{\mathrm{ben}}$Wetted surfacem^2^$\vartheta_{\mathrm{Fl}}$Temperature of the fluid°C$\vartheta_{\mathrm{Wa}}$Temperature of the wall°C$R_{\mathrm{th},\mathrm{conv}}$Thermal resistance for convectionK/W

In convection, a distinction is made between free convection and forced convection. If the body is in a fluid at rest, this corresponds to free convection. This occurs, for example, between the motor housing and the surroundings. Forced convection, on the other hand, describes the actively flowing body. Forced convection, therefore, takes place in the cooling jacket between the housing and the cooling medium. With forced convection, the flow form is of great importance. A distinction is made between laminar and turbulent flow. Laminar flow is an ordered form of flow. In turbulent flow, on the other hand, the flow exhibits turbulence, which occurs in the direction of flow and across it. This means that heat is dissipated more effectively in turbulent flow. The mathematical relationships mentioned apply to both free and forced convection [9]. Heat dissipation through turbulent flow, in particular, significantly increases the complexity of detecting heat propagation in water-cooled EMs. Due to the continuous flow of cooling water around the stator, the heat generated inside is dissipated directly with the cooling water by forced convection. In addition, the stator is separated from the housing surface by an additional casing. This means that the losses generated inside the EM in the form of heat on the housing surface of the EM are difficult to measure. If a hotspot occurs inside the motor, this initially goes unnoticed and can lead to bearing damage, for example. Thermal monitoring of EMs on test benches is usually carried out using type K thermocouples. These are attached to the surface of the motor housing and measure the surface temperature. However, only a few measuring points are involved here. It is, therefore, not possible to detect hotspots. Potential damage may, therefore, not be detected in time or at all. By using thermography, a significantly larger proportion of the engine housing can be monitored for potential temperature increases. Each pixel corresponds to a measuring point, which means that 307,200 measuring points are possible and thus, the housing surface of the engine can be thermally recorded.

### 2.2. Basics of Infrared Thermography

Infrared thermography or thermal imaging is a non-destructive examination method. It makes it possible to observe heat patterns on an object’s surface. Any object with a temperature above absolute zero (i.e., T > −273.15 °C) emits electromagnetic radiation. The infrared range of the electromagnetic spectrum comprises wavelengths in the range of 0.75–1000 µm. This radiation can be visually traced using infrared sensors [13]. With the help of these sensors, infrared measuring devices are able to detect the infrared radiation emitted by an object and convert it into an electronic signal [2]. Infrared radiation is the energy that is emitted from the surface of an object whose temperature is above absolute zero. The emitted radiation is dependent on the temperature of the material. The higher the temperature of the material, the greater the intensity of the emitted infrared energy. The radiant energy that hits an object can be dissipated in three ways: absorption, transmission and reflection [2]. The respective proportions of the total radiant energy of a body are referred to as absorptivity, transmittance and reflectivity. Three parameters are taken into account here: the spectral absorption $\alpha_{\lambda }$, the spectral reflectance $\rho_{\lambda }$ and the spectral transmittance $\tau_{\lambda }$. These parameters are dependent on the wavelength. As in Equation (3), the sum of these parameters must be one at any given wavelength [2]:

(3) $$\alpha_{\lambda }+\rho_{\lambda }+\tau_{\lambda }=1$$

In the case of opaque materials, the radiation is merely absorbed or reflected, resulting in Equation (4) [2]:

(4) $$\alpha_{\lambda }=1-\rho_{\lambda }$$

Materials are described as black bodies if their transmittance and reflectivity are zero. In this case, the entire radiation energy is absorbed, resulting in Equation (5) [2]:

(5) $$\alpha_{\lambda }=1$$

The electromagnetic radiation of a black body $W_{\lambda b}$ can be calculated as in Equation (6) using Planck’s law [2]:

(6) $$W_{\lambda b}=\frac{C_{1}\;\lambda^{-5}}{e^{\frac{C_{2}}{\lambda T}}\;-1}$$

$C_{1}$ and $C_{2}$ are constants, λ corresponds to the wavelength and T to the temperature. The wavelength emitted by electromagnetic radiation depends on the temperature. The higher the temperature of the object, the shorter the emitted wavelength [2].

The emissivity $\varepsilon_{\lambda }$ of an object for a wavelength λ is defined by the ratio of the radiant energy $W_{\lambda }$ emitted by the object to the radiant energy of a black body $W_{\lambda b}$ at the same temperature. As shown in Equation (7) [2]:

(7) $$\varepsilon_{\lambda }=\frac{W_{\lambda }}{W_{\lambda b}}$$

Accordingly, emissivity plays an important role in temperature measurement using infrared and serves as a calibration parameter. It indicates how much radiation is emitted by the object compared to a black body. Materials with a low emissivity emit less infrared radiation at the same temperature than materials with a high emissivity [2].

### 2.3. Thermography on Electric Machines

The diagnosis of faults in EMs using thermography has been used for a number of years. Thermography is of particular interest for detecting damage to EMs on test benches due to its non-contact temperature monitoring. Another reason for the use of thermography on test benches is the large number of measuring points. This allows for not only the thermal monitoring of individual components but also the entire test bench structure.

The following is a brief overview of previously published work on damage detection for air-cooled EMs on test benches using thermography. Work on offline damage detection as well as online damage detection is presented.

In the work by Garcia-Ramirez et al. [3], a method is presented in which thermal images are used to diagnose faults in a kinematic chain. The kinematic chain analysed consisted of the shaft of the air-cooled induction motor, the coupling at the load and the bearing at the drive end. The test assembly consisted of an air-cooled 1492 W three-phase induction motor coupled to a gearbox with a transmission ratio of 4:1. The gearbox drives a permanent magnet motor. The thermal images were recorded with the FLIR G320 thermographic camera (Wilsonville Oregon). The measurement duration was 60 min in each case, with the camera generating an image of the entire kinematic chain every minute. Four different load cases were analysed in this work. Firstly, thermal images of the intact state were generated. In the second load case, bearing damage was simulated by drilling a hole with a diameter of 1.191 mm in the outer ring of the bearing. In the third load case, an imbalance was provoked by attaching a screw to the shaft coupling of the induction motor. The fourth load case simulated an alignment error of the shafts. The regions of interest in the generated infrared images were then characterised using statistical features. Finally, the authors used a distributed, self-organising map structure to modulate the nominal heat distribution so that they could perform fault detection and identification.

The work of Morales-Perez et al. [14] presents a method based on the analysis of a specific area in infrared images for the detection of bearing damage in air-cooled induction motors. The test setup consisted of a three-phase induction motor, a CFW-08 frequency converter from WEG and the FLIR E60 thermal imaging camera, which was positioned at a distance of 1 m from the EM. Three different load cases were analysed. On the one hand, the intact condition was used to generate reference images and, on the other hand, defects on the outer raceway and on the bearing cage. The raceway defect was simulated with a cross-section of 2.15 mm depth and 1.2 mm width on the bearing. The discontinuity of the outer raceway was thus achieved. The cage defect was visualised with a deformation of the component, which was intended to simulate incorrect handling during bearing installation. The EMs were then run for 180 min at half load while video recordings were generated with the thermal imaging camera. The generated videos were analysed using the FLIR ResearchIR MAX 4.40 software, which allowed the temperatures at the defined areas to be extracted. Assuming that the temperature does not change abruptly, the videos were generated at a frame rate of 30 FPS and one frame was analysed every minute. Three areas on the motor housing were selected for the analysis according to their temperature change. A measuring point directly on the bearing was selected for the first area. The second area was located at a thermal transition area. The third measuring point was placed near the fan. The second and third areas were thus determined as a temperature reference in order to evaluate the heat propagation at the bearing. Based on these analysed areas, bearing damage could be determined with the proposed method using the infrared images.

In a paper by Singh and Naikan [15], an infrared thermography-based diagnosis of inter-turn faults and cooling system failures in three-phase induction motors, an algorithm for diagnosing inter-turn faults and failures of the cooling system using infrared is presented. The FLIR E60 camera was used to monitor the motor surface temperature. The images were taken from a fixed distance. Infrared images were generated of a faultless engine and a damaged engine. A ventilation failure was simulated for the damaged engine. For this purpose, a piece of paper was glued to the side of the motor fan so that a cooling failure could be simulated. The recorded infrared images were then thermally analysed using the FLIR tools 5.13 software and Matlab for misdiagnosis. An algorithm is proposed that utilises thermography to effectively diagnose engine faults and reduce fault interpretation.

Another paper by Redon et al. [16] deals with the development of a diagnostic tool based on deep learning algorithms and infrared images for the condition monitoring of induction motors. In the test setup, a 4-pole 1.1 kW laboratory induction motor was driven by a DC machine. A FLIR S65 infrared camera connected to a PC was used for the measurements. The camera measurements were taken over the entire transient process, from the motor’s standstill to stationary driving. The ThermalCAM Researcher 2.10 PRO software was used to capture and analyse the infrared images. The infrared images were recorded at a sampling rate of 1 Hz. The software makes it possible to precisely analyse the temporal development of the temperature distribution on the engine frame and to export the images for subsequent analysis. In this work, several load cases for damage to the motor were analysed. The tests were carried out with the load cases bearing damage, broken shaft, fan damage and imbalance of the stator winding. The bearing damage was caused by a combination of inadequate lubrication and cage damage. The breakage of the shaft was caused by holes in the connection points of the shaft and the short-circuit end rings. The failure of the fan was simulated by a piece of cardboard that completely blocked the airflow into the motor. For the load case of stator winding imbalance, a high-impedance connection point was simulated by connecting an external resistor in series with one of the supply phases. The thermal imaging camera recorded videos from all tests. The infrared images were extracted from each recorded video using Matlab and then processed in a deep learning model. The classifier presented in this paper is based on a deep convolutional neural network. The classifier was trained on each of the four load cases. Under stationary conditions, up to 100% accuracy was achieved.

The work by Mahami et al. [17] presents a non-contact and non-intrusive method for monitoring and diagnosing faults in a three-phase induction motor using thermography. For the experiments, infrared images of an intact induction motor and an induction motor with eight different short-circuit faults in the stator windings were generated. The measurements were carried out with a Dali-tech T4/T8 infrared thermal imaging camera. In this work, the Bag-of-Visual-Word method is used to extract features from the thermal images. Subsequently, different fault patterns in the engine were automatically identified using an extremely randomised tree. This achieved a classification accuracy of 100%.

In another paper by Kahanjani and Ezoji [18], electric fault detection in three-phase asynchronous motors was investigated using deep network-based features of thermograms. An air-cooled, three-phase induction motor was used to generate the infrared images. Normal operation and the load cases of rotor blockage, cooling fan blockage and short circuit faults in the stator winding were analysed. The region of interest (ROI) in the thermograms was extracted from the infrared images using the Scale-invariant feature transform (SIFT)-based algorithm. The SIFT algorithm is used to detect and describe local features in images. Subsequently, the extracted ROIs were processed by pre-trained Convolutional Neuronal Networks. The training areas were divided into two cold and hot clusters by K-Means. The training data were classified using the Support Vector Machine (SVM) algorithm. The SVM algorithm serves as a classifier and divides the objects into classes so that the largest possible area around the class boundaries is free of objects. The proposed method achieved 100% accuracy of induction motor fault detection.

In the papers listed here, some methods were presented that enable damage detection using thermography on EMs. However, these are methods that are carried out on air-cooled EMs. Air cooling means that the motor has no additional cooling jacket. The heat generated by the damage can be dissipated directly via the surface, which can be recognised on the infrared images. This makes it easy to recognise hotspots where they have occurred. The work presented makes it clear that thermography is a tool for detecting damage to air-cooled EMs.

The EMs on drivetrain test benches, on the other hand, are water- or oil-cooled. They have a cooling jacket. This additional jacket and the cooling water prevent the temperature from being measured directly on the engine surface. In addition, the heat is dissipated through the cooling jacket in the cooling water by forced convection. Accordingly, the heat transfer mechanisms inside the machine are strongly influenced by the cooling jacket [9]. The temperature on the surface of the motor housing, therefore, does not allow any conclusions to be drawn about hotspots inside the motor. This makes it difficult to understand the thermal behaviour of water-cooled EMs. In this paper, a method is presented in which the already frequently used condition monitoring technique of thermography is now to be applied to water-cooled machines. This method makes it possible to recognise hotspots of EMs despite a cooling jacket. The aim is to demonstrate the potential of thermography as a valuable tool for detecting faults in water-cooled EMs.

## 3. Materials and Methods

The following section presents a method for monitoring the thermal condition of water-cooled EMs using thermography. The aim of the method is to generate initial findings on the use of thermography on water-cooled EMs. In addition, initial measurements provide results that can be used to create an AI for monitoring the condition of water-cooled EMs.

**Figure 1:**
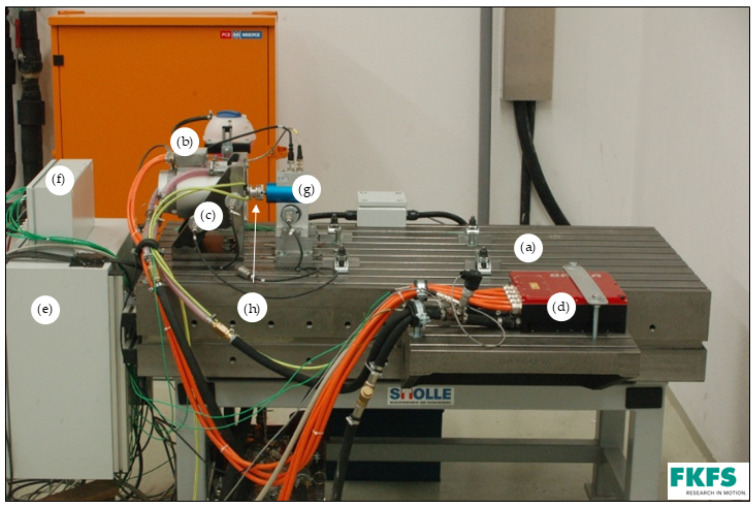
FKFS development test bench for high-speed electric machines: clamping plate (a), EM (b), mounting bracket (c), inverter (d), connection cabinet (e), measuring technology rack (f), torque measuring shaft (g), high-speed clutch (h).

The tests are carried out on a drive test bench at the Research Institute for Automotive Engineering and Powertrain Systems Stuttgart (FKFS). This is shown in Figure 1. The test bench has a modular design and consists of a vibration-insulated clamping plate (a), the electric drive unit (EM (b), mounting bracket (c) and inverter (d)), as well as a connection cabinet (e) and measurement rack (f). In addition, a torque measuring shaft (g) is installed to which the device under test (DUT) to be tested is mechanically connected. The drive power is transmitted via a metal diaphragm coupling (h) in high-speed design. This compensates for shaft misalignments in the axial and radial directions as well as angular misalignments. The test bench used is a newly installed development test bench for high-speed EMs. On this test bench, the mechanical, thermal and hydro-dynamic properties of EMs can be analysed at very early stages of development. The special feature of the test bench is that the DUT itself does not have to generate any torque for the drive. Instead, the DUT is towed by the test bench machine and, therefore, does not require any electromagnetic components or power electronics. Speeds of up to 24,000 rpm are possible with this machine. With the help of automation technology, measurement data of up to 4 kHz can be generated in MDF 4.0 format in real time. Thanks to automation technology, a fully automated test drive is possible. This enables tests such as ageing properties or cycle testing. Heat input and heat dissipation can be precisely determined using water or oil conditioning.

For the method presented, only the test bench machine is analysed. The machine is mounted on the test bench using a support (mounting bracket). A coupling is mounted on the EM, which serves as a connection between the EM and the torque-measuring hub. The setup is constructed using the radial–axial alignment method so that no alignment errors can occur and unwanted vibrations are avoided. A water-cooled asynchronous machine from Brusa, model ASM2.06.11.W, is used for the test. The EM is supplied with voltage via the Brusa DMC514 inverter. Table 1 shows the performance data of the test bench.

A temperature control unit from Regloplas is used for continuous cooling of the motor at 20 °C. This pumps a glycol–water mixture through the cooling jacket of the EM at a flow rate of 10 litres per minute. The Regloplas can be precisely adjusted. This means that the cooling jacket of the EM is constantly flushed with cooling water at a temperature of 20 °C, regardless of the load and speed. The FLIR A655sc thermal imaging camera is used for the investigations. To adjust the emissivity for the camera, the EM is painted black. The emissivity is then 0.95. Table 2 shows the performance data of the thermal imaging camera.

**Figure 2:**
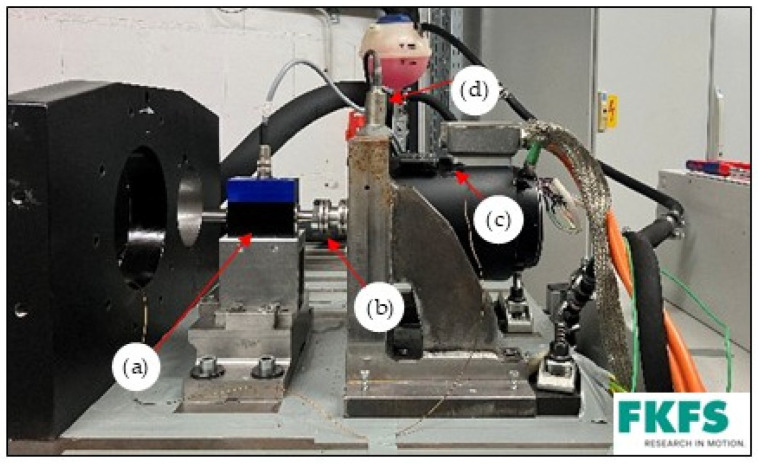
Test bench setup with painted EM: (a) torque measuring hub; (b) coupling; (c) type K thermocouple; (d) vibration velocity sensor.

**Figure 3:**
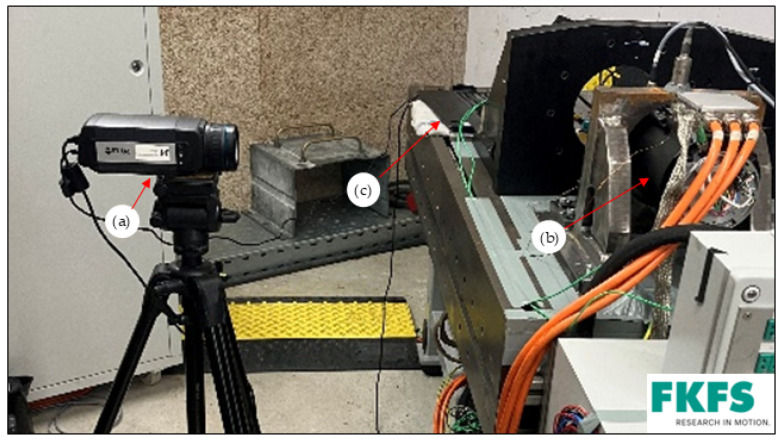
Experimental setup: (a) thermal imaging camera; (b) EM; (c) laptop.

Figure 2 and Figure 3 shows the test bench setup. The thermal imaging camera is positioned next to the test bench machine at a distance of 1 m so that the measuring range covers the entire machine. The thermal imaging camera is connected to a laptop, which records the measurements. In addition, two type K thermocouples are attached to the surface of the motor housing to calibrate the camera. A vibration velocity sensor is mounted on the support to protect the EM and the test bench. This monitors the vibration velocities so that they do not exceed a defined level and serves as protection against mechanical damage to the drive unit and measurement technology caused by excessive vibration velocities.

**Figure 4:**
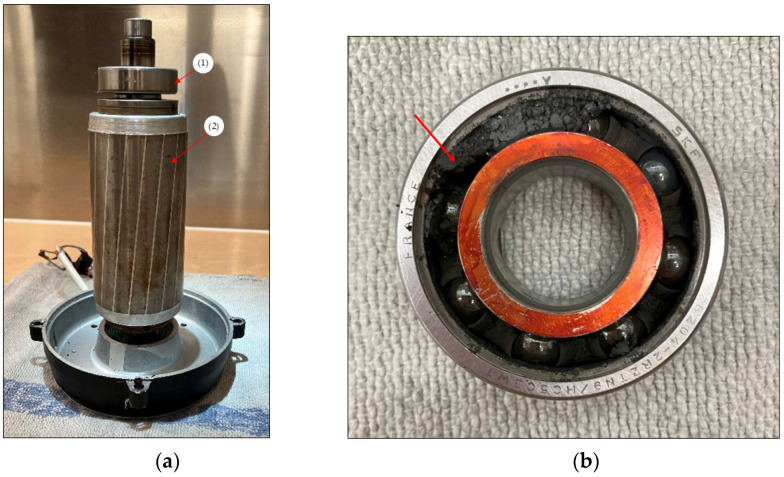
Rebuilding the motor: (**a**) shows the assembly of (1) damaged bearing and (2) rotor; (**b**) shows the bearing damaged by sand.

To generate the infrared images, reference measurements are first carried out with an intact motor and cooling. For the tests with bearing damage, the rotor is dismantled, as shown in Figure 4. The bearing (1) is removed from the rotor (2) and replaced with a damaged bearing. Sand is filled into the bearing, which is intended to synthetically recreate a damaged bearing so that lubrication is no longer provided, as shown in Figure 4b. The bearing is then pressed back onto the rotor and installed in the EM.

The measurements are then carried out with bearing damage and connected cooling. To ensure that the results are reproducible, the measurement time for each test is 45 min. The damaged bearing results in high vibration velocities; so the maximum speed that can be reached is 7500 rpm. In order to increase the friction within the bearing, an oscillating operation was selected for the tests. This means that the speed is constantly varied between 7000 rpm and 7500 rpm. Varying the speed sets the balls in continuous motion within the bearing. The balls are accelerated and decelerated again, which leads to increased bearing friction due to alternating contact between the balls and the cage. The tests are all run according to the same speed sequence.

## 4. Results

**Figure 5:**
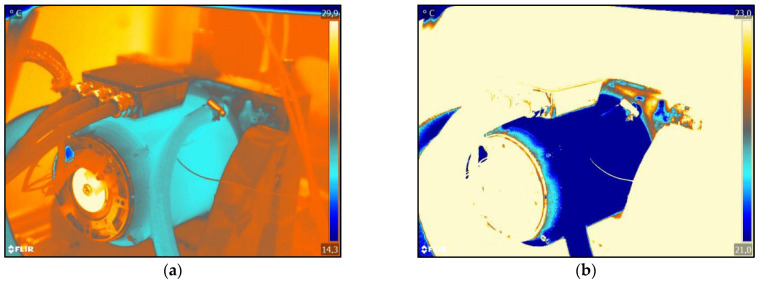
FLIR infrared images of the intact EM as reference: (**a**) shows the processed infrared image with the environment; (**b**) shows the processed infrared image without the test bench environment.

As a reference for the simulation of the damage, a test with an intact engine and a coolant temperature of 20 °C is first run. The measurement duration is 45 min in each case until stationary thermal behaviour is achieved. A video is generated with a thermal imaging camera over the entire measurement period. The frame rate of the measurements is 6 FPS. Infrared images are then extracted from the generated videos using the FLIR Tools 5.13 software. For better visibility of the warmer areas in the image, a pseudo colour palette is applied to the image. The Arctic setting of the FLIR Tools software is used for this. This setting makes it possible to generate a coloured image from a black and white image so that the temperature differences are visualised, as can be seen in Figure 5a.

The infrared images show a clear difference in colour between the surroundings and the motor. The blue pixels represent the cooler area and the red pixels the warmer area. The tests were carried out in summer so that the ambient temperature was higher than the surface temperature of the motor housing, which resulted in the colouring of the infrared images. Due to the flow of coolant through the casing, the power loss inside the motor in the form of heat cannot be recognised on the surface of the casing, as can be seen in Figure 5a. It can also be seen that the cooling of the jacket is evenly transferred to the support in which the motor is located. The cooling of the motor is, therefore, so effective that it makes the heat flow of the motor unrecognisable to the human eye. Based on these infrared images, the test bench engineer cannot recognise any damage to the engine with the human eye. This means that possible bearing damage would go unnoticed. However, in order to make possible bearing damage visible to the test bench engineer, the infrared images must be further processed.

For a more precise analysis, the images are processed again using the FLIR Tools software so that the region of interest (ROI) of the engine is extracted and the surroundings are no longer visible (see Figure 5b).

**Figure 6:**
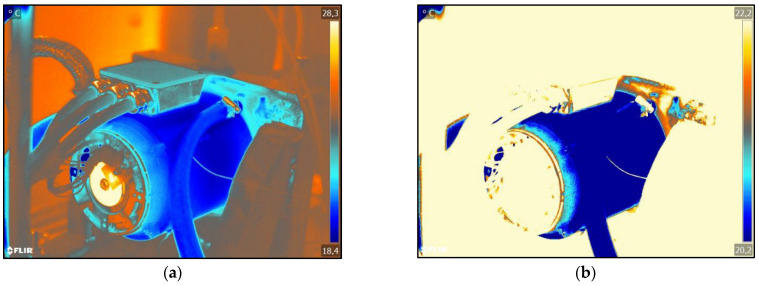
FLIR infrared images of the damaged engine: (**a**) shows the processed infrared image; (**b**) shows the processed infrared image without the test bench environment.

In Figure 5b, the motor is cut free from its surroundings. The same procedure is now carried out on the damaged motor, as shown in Figure 6.

Figure 5b and Figure 6b show that the heat propagation along the end shield is now visible. The friction losses of the bearing are transferred to the end shield in the form of heat, which is shown here by the red pixels. In a direct comparison of Figure 5b and Figure 6b, however, no significant differences can be recognized; therefore, the bearing damage cannot be determined. Without further analysis using the software, the difference between the intact and the damaged engine is not apparent to the test bench engineer. The difference in colour in the individual pixels cannot be seen with the human eye. However, as each pixel is assigned a temperature, each pixel must be analysed separately in order to determine the temperature difference.

**Figure 7:**
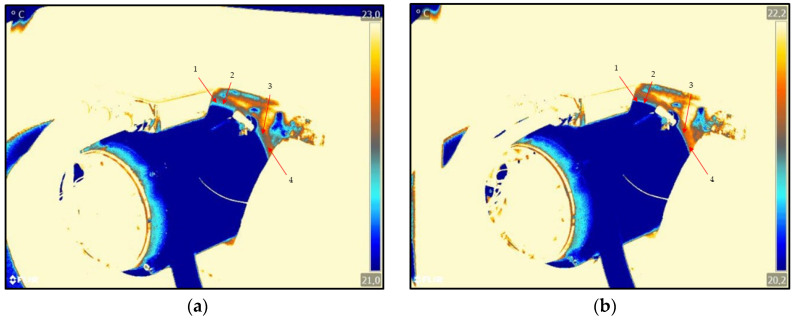
FLIR infrared images of the motor in comparison: (**a**) shows the intact motor; (**b**) shows the motor with bearing damage.

In order to visualise the temperature differences and the resulting temperature curve, the infrared images must be further analysed. Figure 7 shows the infrared images of the extracted EMs again. As the measurements were taken on different days, the ambient temperature was different. This leads to the different maximum temperatures of the individual measurements. The heating along the end shield is marked by arrows in Figure 7b. In this area, hotspots are visible through the red pixels and serve as a basis for further analysis.

**Figure 8:**
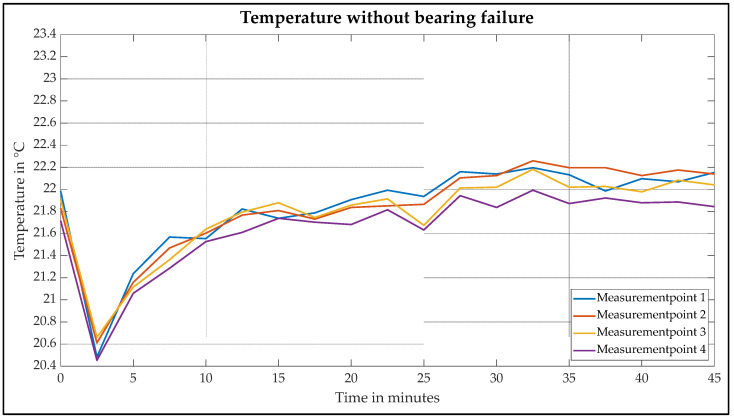
Temperature curve of the intact motor.

**Figure 9:**
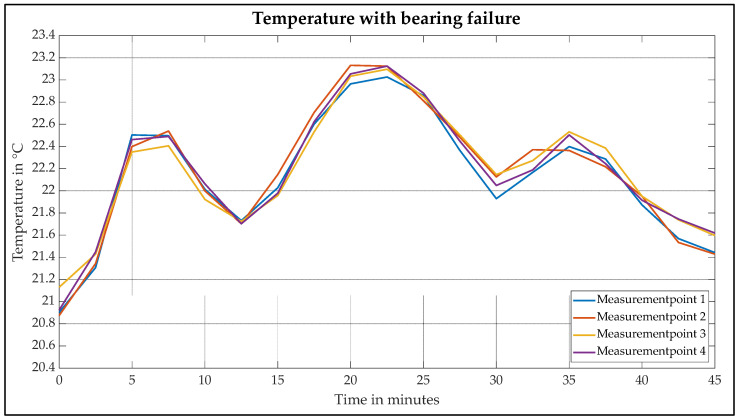
Temperature curve of the damaged motor with bearing damage.

From the infrared images in Figure 7a,b, four measuring points are determined in the area of the hotspots on the end shield for further analysis. The measuring points are determined in such a way that they are located on the end shield and have the same distance from each other. The measuring points are marked by the arrows in Figure 7a. These points are analysed when measuring the intact motor and when measuring the motor with bearing damage. For this purpose, the temperature change of each measuring point is analysed every 2.5 min over the entire measuring time of 45 min. Figure 8 and Figure 9 show the temperature changes of the four measurement points over the entire measurement period.

Figure 8 shows the temperature curve of the intact motor. The abscissa represents the time course of the measurement over 45 min. The ordinate describes the measured temperature. A temperature of 21.8 °C is determined at the start of the measurement. As the ambient temperature is greater than 20 °C over the entire measuring range and the motor housing is warmed up by the ambient temperature, the temperature initially drops to 20.5 °C due to cooling. The temperature of all measuring points then begins to rise.

The curve of the measurements is influenced by two main factors. On the one hand, heat development, which is caused by friction, copper losses and iron losses, leads to an increase in temperature. Secondly, the continuous cooling by the circulating cooling water leads to heat dissipation and thus to a decrease in temperature. These factors result in a curve characterized by high and low points. This curve can be seen at each of the four measuring points. However, an increase in temperature can be identified in the individual measurements over the entire measurement time. After 30 min, an almost thermally stationary state between 21.9 °C and 22.2 °C is reached at all four measuring points.

Table 3 shows the measurement series of the intact motor again for clarification and is given to two decimal places.

Figure 9 shows the temperature curve of the motor with bearing damage. The abscissa shows the course of the measurement over 45 min. The ordinate describes the measured temperatures. In contrast to the intact motor, however, the temperature does not rise continuously until thermal equilibrium is reached but fluctuates over the course of the measurement. The curves of the four measuring points are very similar. For this reason, the curve of the first measuring point is described in more detail below as an example.

At the start of the measurement, the temperature rises to 22.5 °C within the first 5 min, which corresponds to a gradient of $\Delta T=0.32\;\frac{\text{°}C}{\min}$. The temperature then drops to 21.7 °C with a gradient of ΔT = 0.16 $\frac{\text{°}C}{\min}$. After 22.5 min, the maximum temperature of 23.1 °C is reached with a gradient of ΔT = 0.14 $\frac{\text{°}C}{\min}$. The temperature then drops again to 21.9 °C with a gradient of ΔT = 0.16 $\frac{\text{°}C}{\min}$. The temperature then rises with a gradient of ΔT = 0.1 $\frac{\text{°}C}{\min}$ to a temperature of 22.4 °C. While thermal equilibrium has already been reached in the measurement of the intact motor, the temperature of the damaged motor drops again to 21.5 °C after 35 min of measurement.

The basic temperature behaviour can be explained in the same way as for an intact motor. Heat dissipation due to power loss causes an increase in temperature, whereas cooling leads to a decrease in temperature. However, the high and low points in the curves of the damaged motor are much more pronounced than in the intact motor. The sand in the bearing leads to the stick-slip effect. This results in an increased short-term frictional torque, which causes a sudden increase in temperature. If the frictional torque is reduced again, the temperature drops again due to the jacket cooling. This pronounced curve can be recognised in each of the four measurements. Table 4 shows the series of measurements of the damaged motor again for clarification.

**Figure 10:**
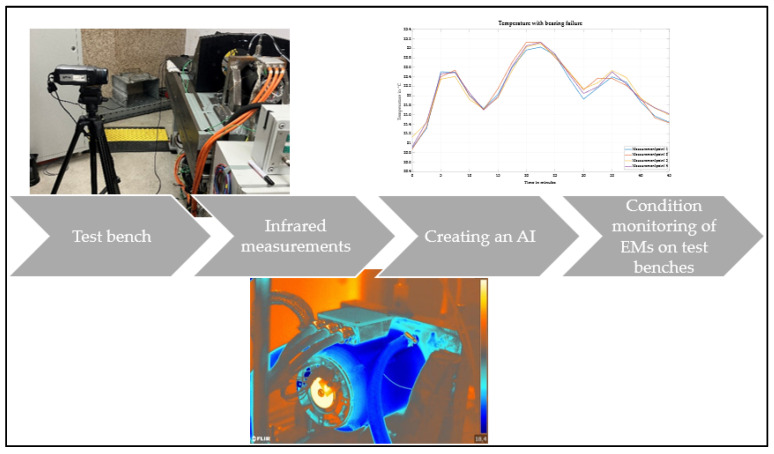
Possible continuation for the development of AI-based condition monitoring.

The different temperature gradients and the fluctuating temperature behaviour of the damaged motor provide sufficient information to distinguish the intact motor from the damaged one. Accordingly, the measurements show that thermography is also suitable for detecting bearing damage in water-cooled EMs. Due to the large number of measuring points, thermography is able to determine the temperature changes despite the heat dissipated by the cooling jacket. The findings of the measurements serve as a basis for condition monitoring, as shown schematically in Figure 10 and described below.

The knowledge gained from this work can serve as a basis for subsequent work. In the field of powertrain testing, the online monitoring of water-cooled EMs is of particular interest. AI for image recognition can be a helpful tool for the test bench engineer. Semantic segmentation can be used to assign warmer pixels to the AI so that hotspots are recognised at an early stage before mechanical damage occurs. The AI can, therefore, be used for early damage detection and alert the test bench engineer to incipient damage in good time. Offline condition monitoring, as presented in this paper, would, therefore, no longer be necessary and would save the test bench engineer additional work.

## 5. Discussion

This paper presents a method for detecting bearing damage in water-cooled EMs based on the analysis of thermographic images. Tests are carried out on a drive test bench, whereby the drive machine represents the machine to be analysed. This has a cooling jacket through which cold cooling water at 20 °C flows continuously. This machine is analysed during operation with the aid of a thermal imaging camera. Each measurement lasts 45 min. Reference measurements are first carried out with an intact EM. The bearing is then damaged by introducing sand and the tests are repeated. During each test, a video is generated using a thermal imaging camera. Infrared images are later generated from the videos produced. These are processed using software so that the heating of the EM can be recognised in the images. Hotspots can be recognised along the end shield, which dissipates the friction losses in the form of heat from the inside of the bearing. Four measuring points along the end shield are determined for further analysis. The measuring points are then displayed in a temperature–time curve diagram. These diagrams show that the temperature in the intact motor rises continuously to a maximum temperature of 22.3 °C during operation due to friction, copper losses and iron losses. A thermal equilibrium is reached after approx. 30 min. The temperature curve of the EM with bearing damage, on the other hand, fluctuates. The temperature rises from 20.9 °C to 22.5 °C directly at the start of the measurement with a gradient of ΔT = 0.32 $\frac{\text{°}C}{\min}$. After a measurement period of 7.5 min, however, it falls again with a gradient of ΔT = 0.16 $\frac{\text{°}C}{\min}$. After 22 min, the measurement reaches its maximum temperature of 23.1 °C. No thermal equilibrium is reached during this measurement; the temperature fluctuations persist over the entire measurement time. This can be attributed to the stick-slip effect. This results in a short-term increase in frictional torque in the bearing, which causes a sudden rise in temperature. If the frictional torque is reduced again, the jacket cooling ensures a reduction in temperature. Accordingly, the tests carried out show that thermal imaging cameras are suitable for diagnosing bearing damage on electric machines with a water-cooled jacket. Despite the cooling jacket, which influences the heat transfer mechanisms within the motor, thermography is able to visualise temperature changes. The results serve as a basis for future work to develop an AI that distinguishes intact motors from damaged ones. This AI could be helpful for test bench engineers when testing EMs on powertrain test benches in the field of online condition monitoring.

## Figures and Tables

**Table 1:** Performance data of the test bench.

Parameter	Value	Unit
Nominal Power	7.5	kW
Maximal Power	35	kW
Norminal torque	6	Nm
Maximum torque	14	Nm
Nominal speed	12,000	rpm
Maximum speed	24,000	rpm

**Table 2:** Performance data of the FLIR A655sc camera.

Parameter	Value	Unit
Image size	640 × 480	Pixel
Temperature measuring points	307,200	-
Temperature range	−20 to +650	°C
Thermal sensitivity	<0.05	°C

**Table 3:** Measuring points of the intact motor in °C.

	5 min	10 min	15 min	20 min	25 min	30 min	35 min	40 min	45 min
1	21.06	21.52	21.74	21.68	21.63	21.84	21.87	21.88	21.84
2	21.12	21.64	21.88	21.86	21.68	22.02	22.02	21.98	22.04
3	21.16	21.60	21.80	21.84	21.87	22.13	22.20	22.13	22.14
4	21.26	21.56	21.74	21.91	21.94	22.14	22.13	22.10	22.15

**Table 4:** Measuring points of the damaged motor with bearing damage in °C.

	5 min	10 min	15 min	20 min	25 min	30 min	35 min	40 min	45 min
1	22.50	22.01	22.03	22.97	22.86	21.93	22.40	21.87	21.44
2	22.40	22.00	22.15	23.13	22.81	22.13	22.36	21.95	21.43
3	22.35	21.92	21.96	23.03	22.85	22.15	22.53	21.95	21.60
4	22.46	22.06	21.98	23.06	22.88	22.05	22.50	21.91	21.62

## Data Availability

The original contributions presented in the study are included in the article; further inquiries can be directed to the corresponding author.

## Abbreviations

AI	Artificial intelligence
CNN	Convolutional Neural Network
DUT	Device under test
EM	Electric Machine
FPS	Frames per second
HiL	Hardware in the Loop
ROI	Region of interest
SIFT	Scale-invariant feature transform
SVM	Support Vector Machine
